# Deep Recurrent Neural Networks for Human Activity Recognition

**DOI:** 10.3390/s17112556

**Published:** 2017-11-06

**Authors:** Abdulmajid Murad, Jae-Young Pyun

**Affiliations:** Department of Information Communication Engineering, Chosun University, 375 Susuk-dong, Dong-gu, Gwangju 501-759, Korea; aaymurad@chosun.kr

**Keywords:** human activity recognition, deep learning, recurrent neural networks

## Abstract

Adopting deep learning methods for human activity recognition has been effective in extracting discriminative features from raw input sequences acquired from body-worn sensors. Although human movements are encoded in a sequence of successive samples in time, typical machine learning methods perform recognition tasks without exploiting the temporal correlations between input data samples. Convolutional neural networks (CNNs) address this issue by using convolutions across a one-dimensional temporal sequence to capture dependencies among input data. However, the size of convolutional kernels restricts the captured range of dependencies between data samples. As a result, typical models are unadaptable to a wide range of activity-recognition configurations and require fixed-length input windows. In this paper, we propose the use of deep recurrent neural networks (DRNNs) for building recognition models that are capable of capturing long-range dependencies in variable-length input sequences. We present unidirectional, bidirectional, and cascaded architectures based on long short-term memory (LSTM) DRNNs and evaluate their effectiveness on miscellaneous benchmark datasets. Experimental results show that our proposed models outperform methods employing conventional machine learning, such as support vector machine (SVM) and k-nearest neighbors (KNN). Additionally, the proposed models yield better performance than other deep learning techniques, such as deep believe networks (DBNs) and CNNs.

## 1. Introduction

Human activity recognition (HAR) has recently attracted increased attention from both researchers and industry with the goal of advancing ubiquitous computing and human computer interactions. It has many real-world applications, ranging from healthcare to personal fitness, gaming, tactical military applications, and indoor navigation. There are two major types of HAR: systems that use wearable sensors and systems that use external devices, such as cameras and wireless RF modules. In sensor-based HAR, wearable sensors are attached to a human body and the human activity is translated into specific sensor signal patterns that can be segmented and identified.

The application of deep learning for HAR has led to significant enhancements in recognition accuracy by overcoming many of the obstacles encountered by traditional machine learning methods. It provides a data-driven approach for learning efficient discriminative features from raw data, resulting in a hierarchy from low-level features to high-level abstractions. The strength of deep learning lies in its ability to automatically extract features in a task dependent manner. It avoids reliance on heuristic hand-crafted features and scales better for more complex behavior-recognition tasks.

The widespread use and availability of sensing technologies is generating an ever-growing amount of data, which along with enhanced computation power have contributed to more feasible applications of deep learning methods. These methods can be utilized to extract valuable contextual information from physical activities in an unconstrained environment. Furthermore, many researchers have employed deep learning approaches to build HAR models in an end-to-end fashion, thereby achieving superior performance compared to previous conventional methods. This strategy has been effective in handling more complex human activities and taking advantage of the proliferating data.

In the field of deep learning, there is a growing interest in recurrent neural networks (RNNs), which have been used for many sequence modeling tasks. They have achieved promising performance enhancements in many technical applications, such as speech recognition [1], language modeling [2], video processing [3], and many other sequence labeling tasks [4]. The rationale behind their effectiveness for sequence-based tasks is their ability to exploit contextual information and learn the temporal dependencies in variable-length input data.

In this paper, we propose the use of long short-term memory (LSTM)-based deep RNNs (DRNNs) to build HAR models for classifying activities mapped from variable-length input sequences. We develop architectures based on deep layers of unidirectional and bidirectional RNNs, independently, as well as a cascaded architecture progressing from bidirectional to unidirectional RNNs. These models are then tested on various benchmark datasets to validate their performance and generalizability for a large range of activity recognition tasks. The major contributions of our work are as follows:

1. We demonstrate the effectiveness of using unidirectional and bidirectional DRNNs for HAR tasks without any additional data-preprocessing or merging with other deep learning methods.
2. We implement bidirectional DRNNs for HAR models. To the best of our knowledge, this the first work to do so.
3. We introduce models that are able to classify variable-length windows of human activities. This is accomplished by utilizing RNN’s capacity to read variable-length sequences of input samples and merge the prediction for each sample into a single prediction for the entire window segment.

The remainder of this paper is organized as follows: Section 2 provides a brief review of related works employing deep learning for HAR and Section 3 presents a background overview of RNNs and LSTM. The proposed models and experimental setup are explained in Section 4 and Section 5, respectively. Performance results and comparisons are presented in Section 6. Finally, discussion and analysis are presented in Section 7.

## 2. Related Works

Early work on using deep learning methods in HAR was based on deep belief networks (DBNs) [5], which were built by stacking multiple layers of restricted Boltzmann machine (RBM). Subsequent DBN-based models exploited the intrinsic temporal sequences in human activities by implementing hidden Markov models (HMMs) above the RBM layers [6]. They performed an unsupervised pre-training step to generate intrinsic features and then used the available data labels to tune the model. However, HMMs are limited by their numbers of possible hidden states and become impractical when modeling long-range dependencies in large context windows.

The use of convolutional neural networks (CNNs) for HAR was introduced in [7], but they used a shallow model and only a single accelerometer. Another model in [8] used deep CNNs with only a single accelerometer. A multi-sensor recognition framework was developed by in [9], where a deep CNN model for two accelerometers was proposed. A new multi-channel time series architecture of CNNs was built in [10]. The architecture proposed in [11] was a compact model of shallow convolutional layers applied to the spectral domain of inertial signals. This model was optimized for low-power devices, but it reintroduced the extraction of handcrafted features by using a spectrogram of the input data. The successful implementation of CNNs for HAR is due to their capability for learning powerful and discriminative features, as well as utilizing convolutions across 1-D temporal sequence in order to capture local dependencies between nearby input samples. To capture local dependencies, CNNs use parameter sharing across time—applying the same convolutional kernel at each time segment—and local connectivity—neurons receiving inputs from small groups of input samples—between adjacent layers [12]. However, sharing parameters across time is insufficient for capturing all of the correlations between input samples. Additionally, local connectivity limits the output to a function of a small number of neighboring input samples.

In this work, we propose the use of DRNNs for HAR models in order exploit their internal memories for capturing the temporal dynamics of activity sequences. In contrast to [13], where CNNs and RNNs were used in a unified framework for activity recognition, our models are based only on DRNNs, meaning we avoid the complexity of combining multiple deep learning approaches in a single framework. Additionally, by using only DRNNs, our models are more flexible for classifying variable-length windows, in contrast to the fixed-length windows required by CNNs. Bidirectional DRNNs have been used in many domains, such as speech recognition and text-to-speech synthesis [1,14]. In in this work we propose using them in HAR models.

## 3. Background: Recurrent Neural Networks

### 3.1. Recurrent Neural Networks

**Figure 1:**
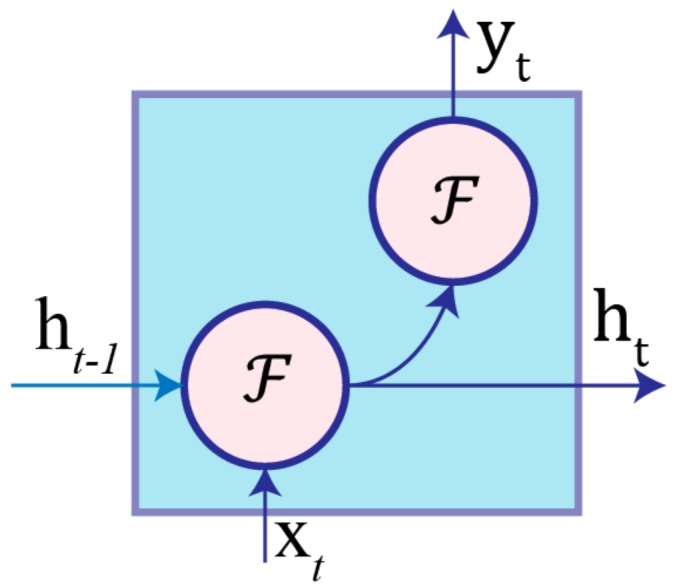
Schematic diagram of an RNN node where $h_{t-1\;}$ is the previous hidden state, $x_{t}$ is the current input sample, $h_{t}$ is the current hidden state, $y_{t}$ is the current output, and ℱ is the activation function.

An RNN is neural network architecture that contains cyclic connections, which enable it to learn the temporal dynamics of sequential data. A hidden layer in an RNN contains multiple nodes. As shown in Figure 1, each node has a function for generating the current hidden state $h_{t}$ and output $y_{t}$ by using its current input $x_{t}$ and the previous hidden state $h_{t-1\;}$ according to the following equations:

(1) $$h_{t}=ℱ\left(W_{h}h_{t-1\;}+\;U_{h}x_{t}+b_{h}\right)$$

(2) $$y_{t}=ℱ\left(W_{y}h_{t\;}+b_{y}\right),$$

where $W_{h}$, $U_{h}$, and $W_{y}$ are the weight for the hidden-to-hidden recurrent connection, input-to-hidden connection, and hidden-to-output connection, respectively. $b_{h}$ and $b_{y}$ are bias terms for the hidden and output states, respectively. Additionally, there is an activation function ℱ associated with each node. This is an element-wise non-linearity function, commonly chosen from various existing functions, such as the sigmoid, hyperbolic tangent, or rectified linear unit (ReLU).

### 3.2. Long Short-Term Memory (LSTM)

Training regular RNNs can be challenging because of vanishing or exploding gradient problems that hinder the network’s ability to backpropagate gradients through long-range temporal intervals [15]. This precludes modeling wide-range dependencies between input data for human activities when learning movements with long context windows. However, LSTM-based RNNs can model temporal sequences and their wide-range dependencies by replacing the traditional nodes with memory cells that have internal and outer recurrence.

**Figure 2:**
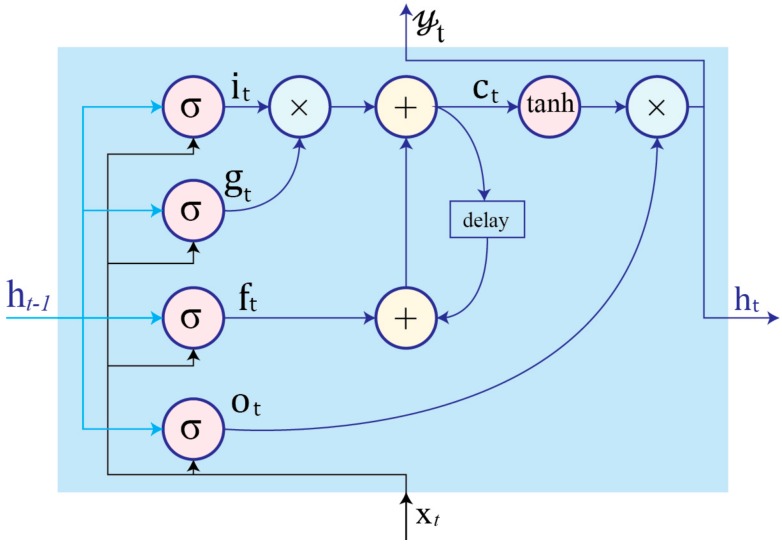
Schematic of LSTM cell structure with an internal recurrence $c_{t}$ and an outer recurrence $h_{t}$. Cell gates are the input gate ${\;i}_{t}$, input modulation gate $g_{t}$, forget gate $f_{t}$, and output gate $o_{t}$. In contrast to an RNN node, the current output ${𝓎}_{t}$ is considered equal to current hidden state $h_{t}$.

A memory cell contains more parameters and gate units, as shown in Figure 2. These gates control when to forget previous hidden states and when to update states with new information. The function of each cell component is as follows:

- Input gate $i_{t}$ controls the flow of new information to the cell.
- Forget gate $f_{t}$ determins when to forget content regarding the internal state.
- Output gate $o_{t}\;$ controls which information flows to the output.
- Input modulation gate $g_{t}$ is the main input to the cell.
- Internal state $c_{t}\;$ handles cell internal recurrence.
- Hidden state $h_{t}$ contains information from previously seen samples within the context window:
  (3) $$i_{t}=\sigma \left(b_{i}+\;U_{i}x_{t}+W_{i}h_{t-1\;}\right)$$
  (4) $$f_{t}=\;\sigma \;\left(b_{f}+\;U_{f}x_{t}+W_{f}x_{t-1}\right)$$
  (5) $$o_{t}=\;\sigma \;\left(b_{o}+\;U_{o}x_{t}+W_{o}h_{t-1}\right)$$
  (6) $$g_{t}=\;\sigma \;\left(b_{g}+\;U_{g}x_{t}+W_{g}h_{t-1}\right)$$
  (7) $$c_{t}\;=\;f_{t}c_{t-1}+g_{t}\;i_{t}$$
  (8) $$h_{t}=\tanh\left(c_{t}\right)o_{t}$$

The training process of LSTM-RNNs is essentially focused on learning the parameters b , U,  and W of the cell gates, as shown in Equations (3)–(6).

## 4. Proposed DRNN Architectures

**Figure 3:**
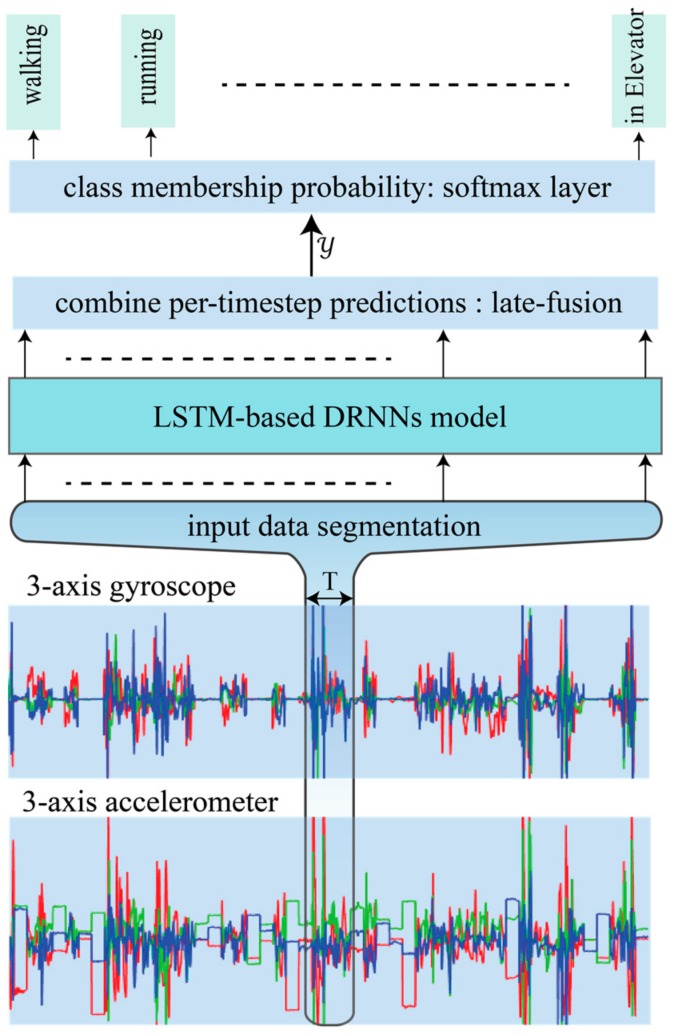
The proposed HAR architecture. The inputs are raw signals obtained from multimodal-sensors, segmented into windows of length T and fed into LSTM-based DRNN model. The model outputs class prediction scores for each timestep, which are then merged via late-fusion and fed into the softmax layer to determine class membership probability.

A schematic diagram of the proposed HAR system is presented in Figure 3. It performs direct end-to-end mapping from raw multi-modal sensor inputs to activity label classifications. It classifies the label of an activity performed during a specific time window. The input is a discrete sequence of equally spaced samples $\left(x_{1},x_{2},\;\ldots ,\;x_{T}\right)$, where each data point $x_{t}$ is a vector of individual samples observed by the sensors at time t. These samples are segmented into windows of a maximum time index T and fed to an LSTM-based DRNN model. The model outputs a sequence of scores representing activity label predictions in which there is a label prediction for each time step $\left({𝓎}_{1}^{L},{𝓎}_{2}^{L},\;\ldots ,\;{𝓎}_{T}^{L}\right)$, where ${𝓎}_{t}^{L}\;\in R^{C}$ is a vector of scores representing the prediction for a given input sample $x_{t}$ and C is the number of activity classes. There will a score for each time-step predicting the type of activity occurring at time t. The prediction for the entire window T is obtained by merging the individual scores into a single prediction. We have used late-fusion technique in which the classification decision from individual samples are combined for the overall prediction of a window. Using the “sum rule” in Equation (9) as the fusion scheme yields better results than other schemes, which is theoretically justified in [16]. We applied a softmax layer over Y to convert prediction scores into probabilities:

(9) $$Y=\;\frac{1}{T}\sum_{t=1}^{T}{𝓎}_{t}^{L}$$

We have developed architectures for three DRNN models, which are as follows:

### 4.1. Unidirectional LSTM-Based DRNNs Model

**Figure 4:**
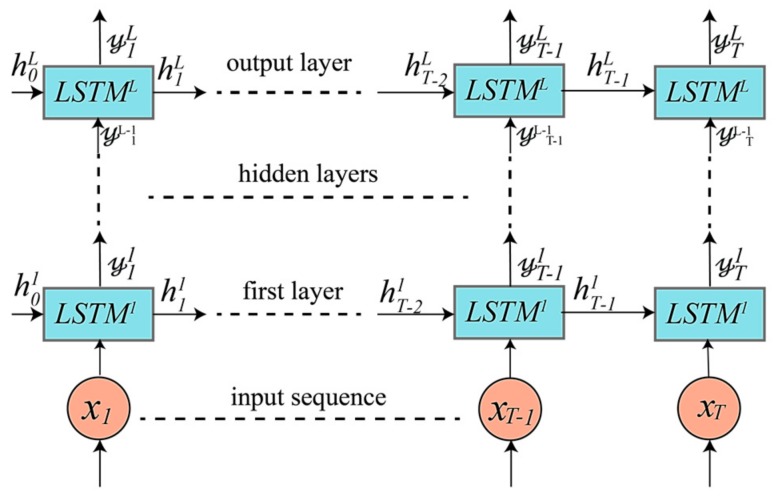
Unidirectional LSTM-based DRNN model consisting of an input layer, several hidden layers, and an output layer. The number of hidden layers is a hyperparameter that is tuned during training.

The first model is built using a unidirectional LSTM-based DRNN, as shown in Figure 4. Using sufficient number of DRNN layers can result in a very powerful model for transforming raw data into a more abstract representation, as well as for learning the temporal dependencies in time series data [1]. The input is a discrete sequence of equally spaced samples $\left(x_{1},x_{2},\;\ldots ,\;x_{T}\right)$, which are fed into the first layer at time t (t=1, 2,…, T).

First, the hidden state $h_{0}^{\ell }$ and internal state $c_{0}^{\ell }$ of every layer ℓ are initialized to zeros. The first layer uses the input sample $x_{t}$ at time t, previous hidden state $h_{t-1}^{1}$, and previous internal hidden state $c_{t-1}^{1}$ to generate the first layer output ${𝓎}_{t}^{1}$ given its parameter $\theta^{1}$ as follows:

(10) $${𝓎}_{t}^{1},\;h_{t}^{1},\;c_{t}^{1}=LSTM^{1}\left(c_{t-1}^{1},\;h_{t-1}^{1},x_{t};\;\theta^{1}\right),$$

where $\theta^{\ell }$ represents the parameters (b,U,W) of the LSTM cells for layer ℓ, as shown in Equations (3)–(6). Any layer ℓ in the upper layers uses the output of the lower layer ${𝓎}_{t}^{\ell -1}$ as its input:

(11) $${𝓎}_{t}^{\ell },h_{t}^{\ell },c_{t}^{\ell }={\mathrm{LSTM}}^{\ell }\left(c_{t-1}^{\ell },{\;h}_{t-1}^{\ell },{𝓎}_{t}^{\ell -1};\;\theta^{\ell }\right).$$

The top layer L outputs $\left({𝓎}_{1}^{L},{𝓎}_{2}^{L},\;\ldots ,\;{𝓎}_{T}^{L}\right)$, which is a sequence of scores representing the predictions at every time step in the window T.

### 4.2. Bidirectional LSTM-Based DRNN Model

**Figure 5:**
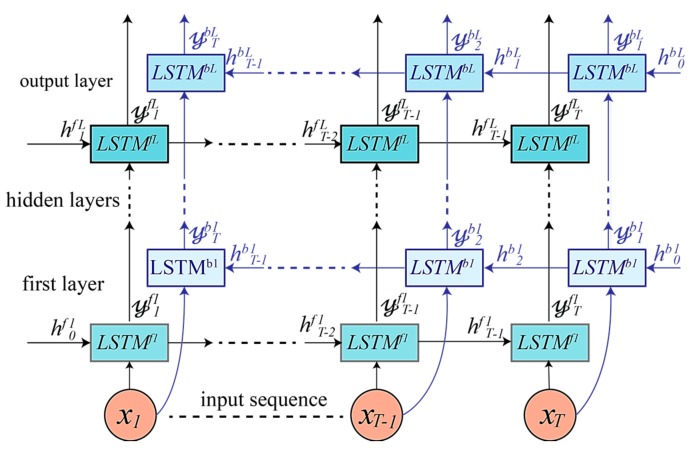
Bidirectional LSTM-based DRNN model consisting of an input layer, multiple hidden layers, and an output layer. Every layer has a forward $LSTM^{f\ell }$ and a backward $LSTM^{b\ell }$ track, and the number of hidden layers is a hyperparameter that is tuned during training.

The second model architecture is built by using a bidirectional LSTM-based DRNN, as shown in Figure 5. It includes two parallel LSTM tracks: forward and backward loops for exploiting context from the past and future of a specific time step in order to predict its label [17]. In the first layer, the forward track $\left(LSTM^{f1}\right)$ reads the input window T from left to right, whereas the backward track $\left(LSTM^{b1}\right)$ reads the input from right to left according to:

(12) $${𝓎}_{t}^{f1},\;h_{t}^{f1},\;c_{t}^{f1}=LSTM^{f1}\left(c_{t-1}^{f1},\;h_{t-1}^{f1},x_{t};\;W^{f1}\right)$$

(13) $${𝓎}_{t}^{b1},\;h_{t}^{b1},\;c_{t}^{b1}=LSTM^{b1}\left(c_{t-1}^{b1},\;h_{t-1}^{b1},x_{t};\;W^{b1}\right).$$

The top layer L outputs a sequence of scores at each time step for both forward LSTM $\left({𝓎}_{1}^{fL},{𝓎}_{2}^{fL},\;\ldots ,\;{𝓎}_{T}^{fL}\right)$ and backward LSTM $\left({𝓎}_{1}^{bL},{𝓎}_{2}^{bL},\;\ldots ,\;{𝓎}_{T}^{bL}\right)$. These scores are then combined into a single vector $Y\;\in R^{C}$ representing classes prediction for the window segment T. The late-fusion in this case will differ from that used in the unidirectional DRNN, Equation (9), because there are two outputs resulting from the forward and backward tracks, which are combined as follows:

(14) $$Y=\;\frac{1}{T}\sum_{t=1}^{T}\left({𝓎}_{t}^{fL}+{𝓎}_{t}^{bL}\right)$$

### 4.3. Cascaded Bidirectional and Unidirectional LSTM-based DRNN Model

**Figure 6:**
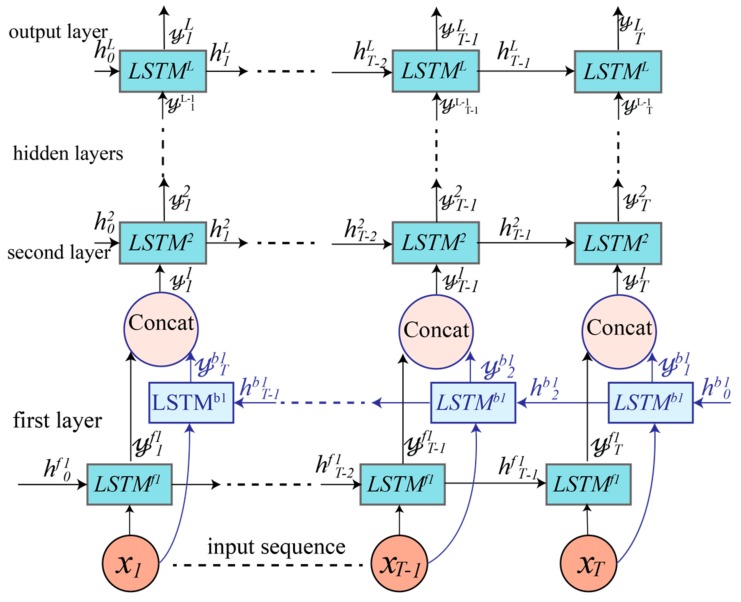
Cascaded unidirectional and bidirectional LSTM-based DRNN model. The first layer is bidirectional, whereas the upper layers are unidirectional. The number of hidden unidirectional layers is a hyperparameter that is tuned during training.

The third model architecture, shown in Figure 6, is motivated by [18]. It is a cascaded structure in which the first layer is a bidirectional RNN and the upper layers are unidirectional. The first layer has a forward LSTM track $LSTM^{f1}$ that generates an output $\left({𝓎}_{1}^{f1},{𝓎}_{2}^{f1},\;\ldots ,\;{𝓎}_{T}^{f1}\right)$ and a backward LSTM track $LSTM^{b1}$ that generates an output $\left({𝓎}_{1}^{b1},{𝓎}_{2}^{b1},\;\ldots ,\;{𝓎}_{T}^{b1}\right)$. These two types of outputs are concatenated to form a new output $\left({𝓎}_{1}^{1},{𝓎}_{2}^{1},\;\ldots ,\;{𝓎}_{T}^{1}\right)$, which is fed into the second unidirectional layer:

(15) $${𝓎}_{t}^{1}={𝓎}_{t}^{f1}+{𝓎}_{T-t+1}^{b1}$$

The upper layers are then treated in the same manner as in the unidirectional model described in Section 4.1.

## 5. Experimental Setup

### 5.1. Datasets of Human Activities

In order to train and evaluate the proposed models, we considered five public benchmark datasets for HAR. The datasets contain diverse movement data, captured by on-body sensors. They contain various activities performed in different environments and are used to validate the applicability and generalization of our models for a large variety of activity recognition tasks. Table 1 summarizes the experimental datasets and the following are brief descriptions of them:

1) UCI-HAD [19]: Dataset for activities of daily living (ADL) recorded by using a waist-mounted smartphone with an embedded 3-axis accelerometer, gyroscope, and magnetometer. All nine channels from the 3-axis sensors are used as inputs for our DRNN model at every time step. This dataset contains only six classes: walking, ascending stairs, descending stairs, sitting, standing, and laying.
2) USC-HAD [20]: Dataset collected by using a high performance IMU (3D accelerometer and gyroscope) sensor positioned on volunteers’ front right hips. The dataset contains 12 basic human activities: walking forward, walking left, walking right, walking upstairs, walking downstairs, running forward, jumping up, sitting, standing, sleeping, in elevator up, and in elevator down. We considered 11 classes by combining the last two activities into a single “in elevator” activity. The reason for this combination is that the model is unable to differentiate between the two classes using only a single IMU sensor. Additional barometer readings are required to determine height changes in an elevator and discriminate between the two classes (up or down in elevator).
3) Opportunity [21]: Dataset comprised of ADL recorded in a sensor-rich environment. We consider only recordings from on-body sensors, which are seven IMUs and 12 3D-accelerometers placed on various body parts. There are 18 activity classes: opening and closing two types of doors, opening and closing three drawers at different heights, opening and closing a fridge, opening and closing a dishwasher, cleaning a table, drinking from a cup, toggling a switch, and a null-class for any non-relevant actions.
4) Daphnet FOG [22]: Dataset containing movement data from patients with Parkinson’s disease (PD) who suffer from freezing of gait (FOG) symptoms. The dataset was built using three 3D-accelerometers attached to the shank, thigh, and lower back of the patients. Two classes (freeze and normal) were considered depending on whether or not the gait of a patient was frozen when the sample was recorded. We used this dataset to train our model to detect FOG episodes in PD patients and prove the suitability of our model for gait analysis using only wearable sensors.
5) Skoda [23]: Dataset containing activities of an employee in a car maintenance scenario. We consider recordings from a single 3D accelerometer, which is placed on the right hand of an employee. The dataset contains 11 activity classes: writing on a notepad, opening hood, closing hood, checking gaps on front door, opening left front door, closing left front door, closing both left doors, checking trunk gaps, opening and closing trunk, and a null-class for any non-relevant actions.

### 5.2. Training

We trained our DRNN models on each dataset using 80% of the data for training and 20% for testing. The weights (parameters) of the models were initialized randomly and then updated to minimize a cost function ℒ. We used the mean cross entropy between the ground truth labels and the predicted output labels as the cost function. The ground truth labels are given in the datasets and indicate the true classes (labels) for the segmented windows. They are provided as a one-hot vector $O\in R^{C}$ with a value $o_{k}$ associated with each class k. The predicted label $\hat{O}\in R^{C}$ contains the probability of every class $p_{k}$ generated by our model:

(16) $$ℒ\left(O,\hat{O}\right)=\;-\sum_{k=1}^{C}o_{k}\;\log p_{k}$$

**Figure 7:**
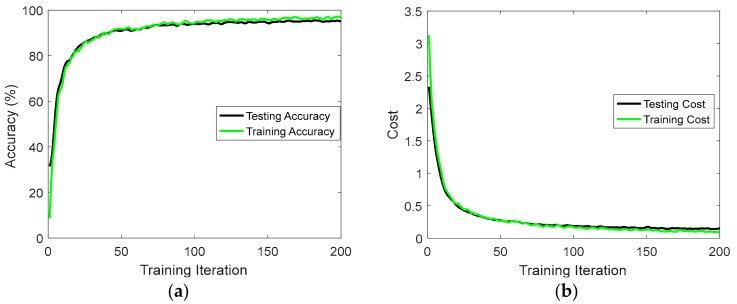
Accuracy and cost of the unidirectional DRNN model for the USC-HAD dataset over mini-batch training iterations: (**a**) training and testing accuracies; (**b**) cross-entropy cost between ground truth labels and predicted labels for both training and testing.

We used an optimization algorithm called Adam that minimizes the cost function by backpropagating its gradient and updating model parameters [24]. Training was conducted on a GPU-based TensorFlow framework in order to utilize the parallel computation power of a GPU [25]. The dropout technique was used to avoid overfitting in our model [26]. Although dropout is typically applied to all nodes in a network, we followed the convention of applying dropout to the connections between layers (not on recurrent-connections or intra-cell connections). The probability of dropping a node during a training iteration is determined by the dropout probability 𝓅, which is a hyperparameter tuned during training and represents the percentage of units to drop. Adopting dropout regularization technique led to a significant improvement in performance by preventing overfitting. Figure 7 presents the accuracy and cost of training and testing processes for the unidirectional DRNN model using the USC-HAD dataset. The gap between training and testing accuracies, as well as the gap between training and testing costs is very small. This indicates that the dropout technique is very effective at forcing the model to generalize and be resilient to overfitting.

During training, the datasets were segmented with different window lengths, as outlined in Table 1. The optimal window length of a dataset depends on the sampling rate and the type of activities performed. We tested various lengths by “trial-and-error” method, then chose the window length that gave better performance results. Training was performed using the raw data without any further data preprocessing or intermediate intervention. The training and testing are generally performed using fixed-length windows, but the inputs of models may be using variable-length windows in the real-time data acquisition scenarios. In real-time application of HAR, data are captured over the course of time and the delay in DRNNs is not fixed. Instead, the network can emit the corresponding label for a variable-length input segment. This is in contrast to other methods, such as CNNs, in which the network must wait until a given fixed-length input segment is complete, before emitting the corresponding label.

### 5.3. Performance Metrics

To verify the performance of the proposed models, we employed four widely used evaluation metrics for multi-class classification [27]:

1) Precision: Measures the number of true samples out of those classified as positive. The overall precision is the average of the precisions for each class:
  (17) $$Per-class\;Precision_{c}=\;\frac{tp_{c}}{tp_{c}+fp_{c}}$$
  (18) $$Overall\;Precision=\frac{1}{C}\left(\overset{C}{\underset{c=1}{\sum }}\frac{tp_{c}}{tp_{c}+fp_{c}}\right),$$
  where $tp_{c}$ is the true positive rate of a class c, $fp_{c}$ is the false positive rate, and C is the number of classes in the dataset.
2) Recall (Sensitivity): Measures the number correctly classified samples out of the total samples of a class. The overall recall is the average of the recalls for each class:
  (19) $$Per-class\;Recall_{c}=\;\frac{tp_{c}}{tp_{c}+fn_{c}}$$
  $$Overall\;Recall\;=\frac{1}{C}\left(\overset{C}{\underset{c=1}{\sum }}\frac{tp_{c}}{tp_{c}+fn_{c}}\right),$$
  where $fn_{c}$ is the false negative rate of a class c.
3) Accuracy: Measures the proportion of correctly predicted labels over all predictions:
  $$Overall\;Accuraccy=\frac{TP+TN}{TP+TN+FP+FN},$$
  where $TP=\overset{C}{\underset{c=1}{\sum }}tp_{c}$ is the overall true positive rate for a classifier on all classes, $TN=\overset{C}{\underset{c=1}{\sum }}tn_{c}$ is the overall true negative rate, $FP\;=\overset{C}{\underset{c=1}{\sum }}fp_{c}$ is the overall false positive rate, and $FN=\overset{C}{\underset{c=1}{\sum }}fn_{c}$ is the overall false negative rate.
4) F1-score: A weighted harmonic mean of precision and recall:
  (22) $$F1\;score=\overset{C}{\underset{c=1}{\sum }}2\left(\frac{n_{c}}{N}\right)\times \;\frac{precision_{c}\times \;recall_{c}}{precision_{c}+recall_{c}}\;,$$
  where $n_{c}$ is the number of samples of a class c and $N=\overset{C}{\underset{c=1}{\sum }}n_{c}$ is the total number of samples in a set with C classes. The F1-score is typically adopted for imbalanced datasets that have more samples of one class and less of another, such as the Daphnet FOG dataset. There are more instances of normal walking (majority class) than of FOG (minority class). The Opportunity dataset is also imbalanced because there are many more instances of the null class than any of the other classes. Using accuracy as a performance metric in imbalanced datasets can be misleading, because any classifier can perform well by correctly classifying the majority class even if it wrongly classifies the minority class.

## 6. Results 

The performance results of our proposed models are presented in this section. The results are compared to other previously introduced methods, which are tested on the same datasets.

### 6.1. UCI-HAD

**Figure 8:**
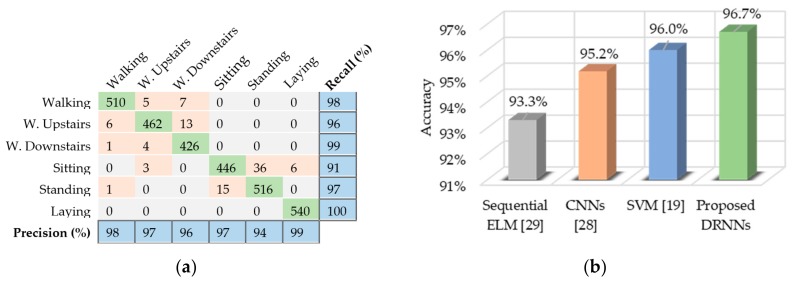
Performance results of the proposed unidirectional DRNN model for the UCI-HAD dataset: (**a**) Confusion matrix for the test set containing the activity recognition results. The rows represent the true labels and the columns represent the model classification results; (**b**) Comparative accuracy of the proposed model against other methods.

For the UCI-HAD dataset, we found that the unidirectional DRNN model with four layers yields best performance results in terms of per-class precision and recall, as shown in Figure 8a. The overall classification accuracy is 96.7%, outperforming other methods, such as CNNs [28], support vector machine (SVM) [19], and sequential extreme learning machine (ELM) [29]. Figure 8b presents a chart of the observed accuracy from our model in comparison with the accuracies achieved by other methods.

### 6.2. USC-HAD

**Figure 9:**
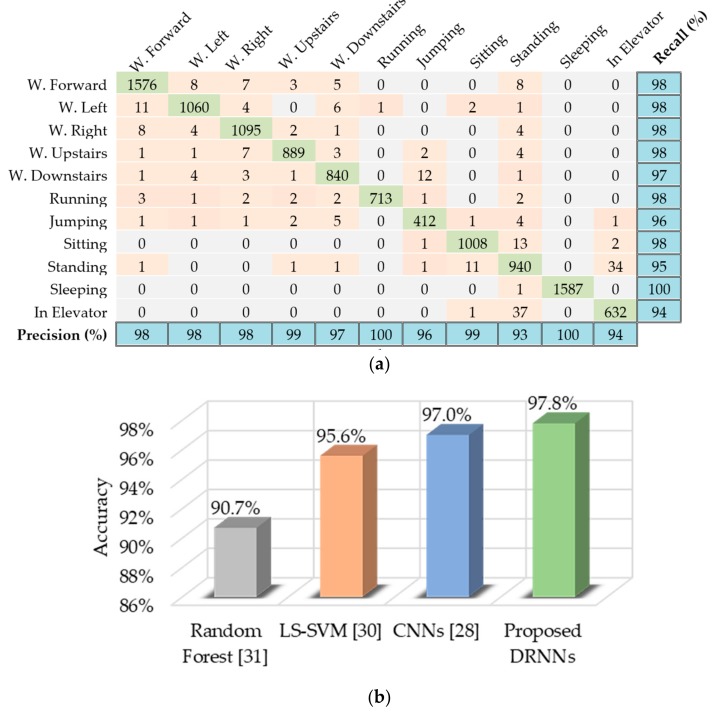
Performance results of the proposed unidirectional DRNN model for USC-HAD dataset: (**a**) Confusion matrix for the test set displaying activity recognition results with per-class precision and recall; (**b**) Comparative accuracy of proposed model against other methods.

We found that the unidirectional DRNN model with four layers yields the best results for the USC-HAD dataset. Figure 9a presents the classification results for the test set in the form of a confusion matrix, along with the per-class recall and precision results. The proposed method achieved better overall accuracy than other methods, such as CNNs [28], least squares support vector machine (LS-SVM) [30], and random forest [31], as shown in Figure 9b.

### 6.3. Opportunity

**Figure 10:**
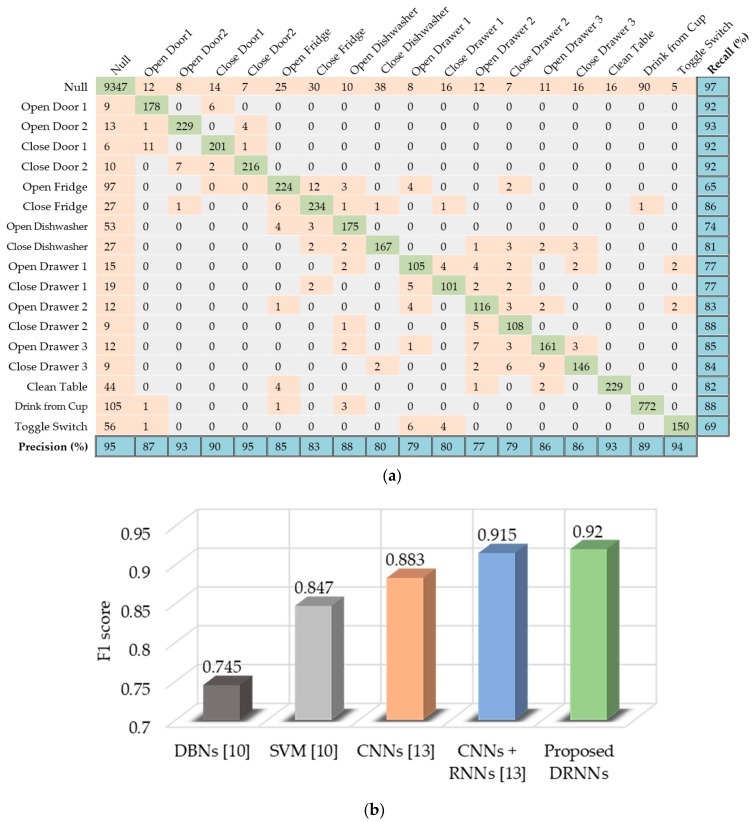
Performance results of the proposed bidirectional DRNN model for the Opportunity dataset: (**a**) Confusion matrix for the test set as well as per-class precision and recall results; (**b**) Comparative F1 score of proposed model against other methods.

The Opportunity dataset is very complex and contains a wide range of activities. Therefore, the bidirectional DRNN model with three layers yields the best performance results. The confusion matrix in Figure 10a summarizes the classification results of the proposed model for the test set, along with the per-class precision and recall results. The proposed method outperforms other methods, such as those based on deep believe networks (DBNs) [10], SVM [10], and CNNs [13]. It also outperformed the state-of-the-art method, which is a combination of CNNS and unidirectional RNNs [13], for the opportunity dataset. Figure 10b presents a performance comparison between the F1 score of the proposed method and those reported by other methods. We used the F1 score as a basis for comparison because the Opportunity dataset is imbalanced, manifested by the dominance of the Null class.

### 6.4. Daphnet FOG

**Figure 11:**
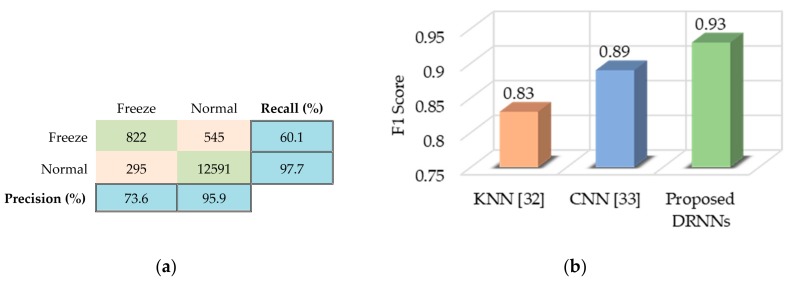
Performance results of the proposed cascaded DRNN model for the Daphnet FOG dataset: (**a**) Confusion matrix for the test set, along with per-class precision and recall; (**b**) F1 score of the proposed method in comparison with other methods.

For the Daphnet FOG dataset, we found that the cascaded DRNN model with one bidirectional layer and two upper unidirectional layers yields the best results. Figure 11a summarizes the classification results for the test set. The low values of recall and precision for the “Freeze” class are caused by the dominance of the “Normal” class. However, our proposed method still outperforms other methods, such as k-nearest neighbors (KNN) [32] and CNNs [33], in terms of F1 score, as shown in Figure 11b.

### 6.5. Skoda

**Figure 12:**
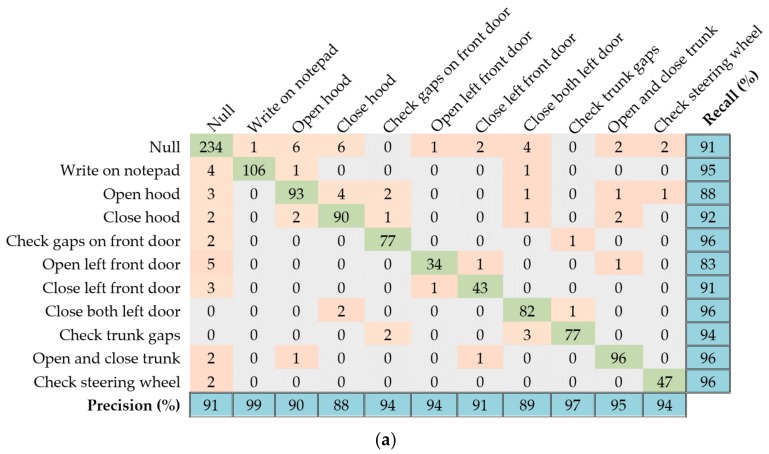

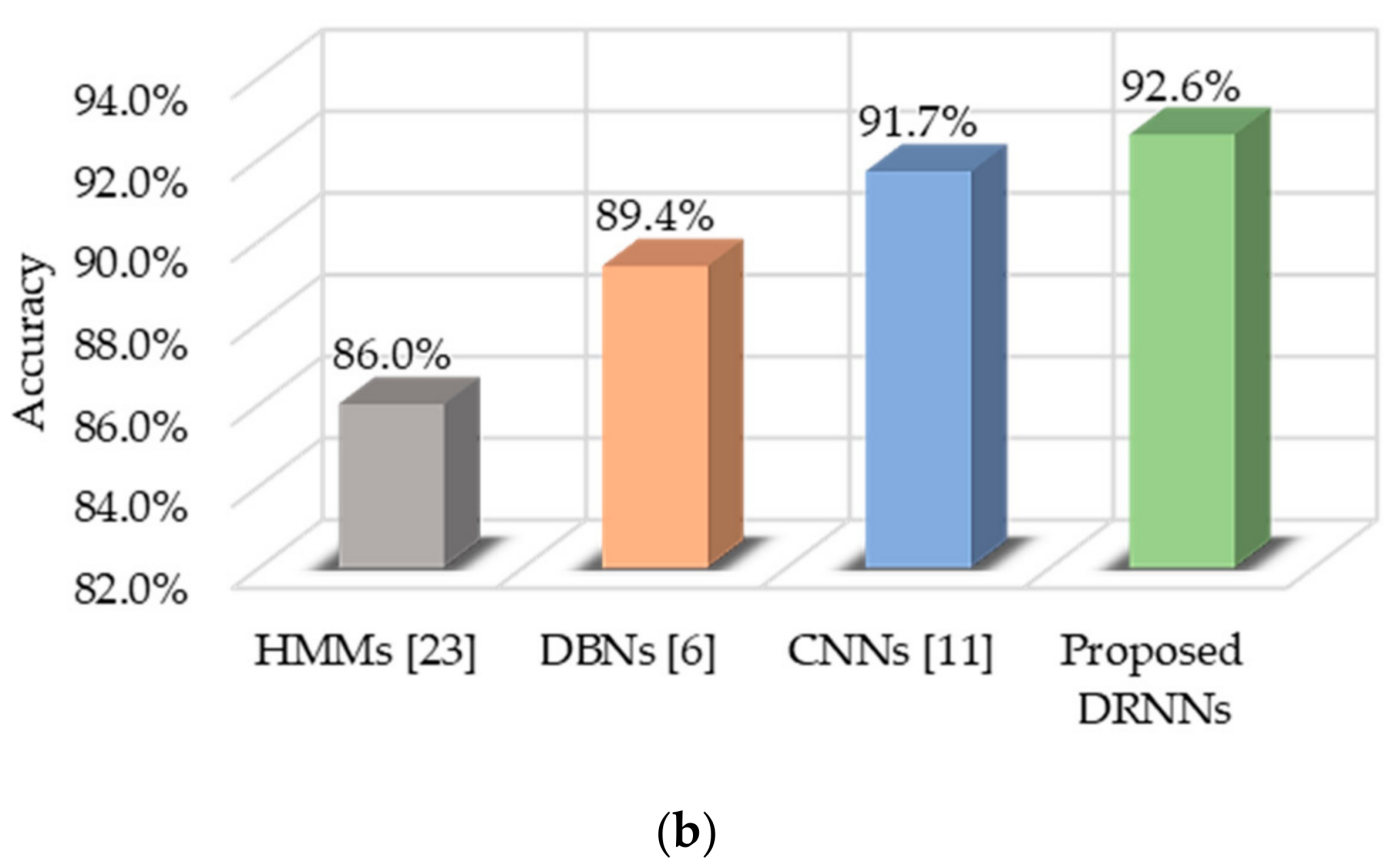
Performance results of the proposed cascaded DRNN model for the Skoda dataset: (**a**) Confusion matrix for the test set as well as per-class precision and recall results; (**b**) Comparative accuracy of proposed model against other methods.

We found that the cascaded DRNN model yields the best results for the Skoda dataset. The model is built using one bidirectional layer and two upper unidirectional layers. Figure 12a presents the classification results for the test set in the form of a confusion matrix, along with the per-class recall and precision results. The proposed method results in an overall accuracy of 92.6%, outperforming other methods such as HMMs [23], DBNs [6], and CNNs [11], as shown in Figure 12b.

## 7. Discussion

The performance results of the proposed models clearly demonstrate that DRNNs are very effective for HAR. All of the architectures performed very well on all of the datasets. These datasets are diverse, which proves that our models are effective for a broad range of activity recognition tasks. The unidirectional DRNN model yielded the best results for the UCI-HAD and USC-HAD datasets, the bidirectional DRNN model gave better results for the Opportunity dataset, and the cascaded DRNN model performed better on the Daphnet FOG and Skoda dataset. Table 2 contains a performance summary for the four datasets.

There are two main reasons for the superb performance of the proposed models for HAR tasks. First, including sufficient deep layers enabled the models to extract effective discriminative features. These features are exploited to distinguish between classified activities and scale up for more complex behavior recognitions tasks. Second, employing DRNNs to capture sequential and time dependencies between input data samples provided a significant improvement in performance compared to other methods.

## 8. Conclusions

We have presented three novel LSTM-based DRNN architectures for HAR tasks. Additionally, we empirically evaluated our models by conducting experiments on four miscellaneous benchmark datasets. Experimental results reveal that the proposed models outperform other state-of-the-art methods. The reason for this improvement in performance is that our models are able to extract more discriminative features by using deep layers in a task-dependent and end-to-end fashion. Furthermore, our models are able to capture the temporal dependencies between input samples in activity sequences by exploiting DRNN functionality. Future work includes experimentation on large-scale and complex human activities, as well as exploring transfer learning between diverse datasets. Investigating resource efficient implementation of a DRNN for low-power devices is also a promising future research direction.

## Figures and Tables

**Table 1:** Summary of human activity datasets used to evaluate the proposed deep learning models. Training window length indicates the number of samples in a window that we found to yield the best results for each dataset. Each dataset was divided into 80% for training and 20% for testing.

Dataset	# of Classes	Sensors	# of Subjects	Sampling Rate	Training Window Length	# of Training Examples	# of Testing Examples
UCI-HAD [19]	6	3D Acc., Gyro., and Magn. of a smartphone	30	50 Hz	128	11,988	2997
USC-HAD [20]	12	3D Acc. & Gyro	14 (5 sessions)	100 Hz	128	44,000	11,000
Opportunity [21]	18	7 IMU sensors (3D ACC, Gyro & Mag.) & 12 Acc.	4 (5 sessions)	30 Hz	24	55,576	13,894
Daphnet FOG [22]	2	3 3D Acc.	10	64 Hz	32	57,012	14,253
Skoda [23]	11	3D Acc.	1 (19 sessions)	98 Hz	128	4411	1102

**Table 2:** Performance summary for the proposed DRNNs on four diverse datasets.

Model	Dataset	Overall Accuracy	Average Precision	Average Recall	F1 Score
Unidirectional DRNN	UCI	96.7%	96.8%	96.7%	0.96
Unidirectional DRNN	USC-HAD	97.8%	97.4.0%	97.4%	0.97
Bidirectional DRNN	Opportunity	92.5%	86.7%	83.5%	0.92
Cascaded DRNN	Daphnet FOG	94.1%	84.7%	78.9%	0.93
Cascaded DRNN	Skoda	92.6%	93.0%	92.6%	0.92
